# Prediction Model of Stress Intensity Factor of Circumferential Through Crack in Elbow Based on Neural Network

**DOI:** 10.1155/2022/8395505

**Published:** 2022-03-18

**Authors:** Xiaohong Li, Xianghui Li, Bin Chen

**Affiliations:** ^1^Liaoning Petrochemical University, Fushun, Liaoning 113001, China; ^2^China University of Petroleum (East China), Qingdao, Shandong 266000, China

## Abstract

Using ANSYS software to establish the finite element model of crack bending tube, the SIF at the tip of the crack is calculated for the difference in the diameter of the pipe, the outer diameter of the elbow, and the bending angle of the bend pipe, and it is used as a neural network to calculate the sample. By using three layers of BP network to establish the prediction model of the SIF of cracked pipe, the simulation of 39 sets of samples proves that the relative error of the BP network model is 0.19% and the mean square error of the network output is 0.0102. The prediction model has high prediction precision and generalization ability and can be used in engineering design and calculation.

## 1. Introduction

Elbows are widely used in engineering structural parts such as building structures, mechanical equipment, and vehicle manufacturing. Defects and cracks are usually inevitable. Tension and bending moment are the load types that pipes bear more. Therefore, it is of great significance to study the fracture parameters of circumferential cracks on the inner wall of elbows under tension and bending moment for the safety assessment of defective structures [1].

Stress intensity factor is an important parameter to control the fracture structure. It is a necessary theoretical basis to determine the service life of crack structure and design crack prevention measures. Fett and Noda [2, 3] used the analytical method to solve the strength factor, but it is only suitable for the relatively simple crack model. Rong et al. [4] recorded a variety of typical crack strength factor calculation formulas, but their mechanical models are infinite and semi-infinite plate structures and bear a single load. For more complex cracks, the classical theory is difficult to give the calculation method of stress intensity factor, which is often solved by numerical methods such as the finite element method, finite difference method, and boundary element method [5]. The finite element method [6] is simple and accurate and has been proved to be an effective method for calculating the stress intensity factor of structures with cracks [7, 8]. Many parameters need to be considered in the strength factor of cracked elbow.

Due to the high complexity and professionalism of the operation of the finite element method, the calculator cannot complete all the calculations of a large number of variables. In this paper, the strength factor training samples are collected by the finite element method, and the artificial neural network method is used. The artificial neural network has strong memory and prediction ability to establish the calculation model of the stress intensity factor of the circumferential through crack of the elbow, so as to realize the calculation of the stress intensity factor of the circumferential through crack of the elbow in a certain range. The results show that the accuracy of the calculation network meets the engineering requirements.

## 2. Calculation of Stress Intensity Factor

The relationship between the stress intensity factor near the crack tip and stress, displacement, and strain is shown in formulas (1)–(3):

(1) $$\begin{array}{c} \overset{\left(N\right)}{\underset{ij}{\sigma }}=\frac{K_{N}}{\sqrt{2\pi r}}\overset{\left(N\right)}{\underset{ij}{f}}\left(\theta \right), \end{array}$$

(2) $$\begin{array}{c} \overset{N}{\underset{i}{U}}=K_{N}\sqrt{\frac{r}{\pi }}\overset{\left(N\right)}{\underset{i}{g}}\left(\theta \right), \end{array}$$

(3) $$\begin{array}{c} \overset{\left(N\right)}{\underset{ij}{\varepsilon }}=\frac{K_{N}}{\sqrt{2\pi r}}\overset{\left(N\right)}{\underset{ij}{h}}\left(\theta \right), \end{array}$$

where *σ*_*ij*_(*i*, *j* = 1, 2, 3) represents the stress component, *u*_*i*_(*i* = 1, 2, 3) represents the displacement component, *N* = I, II, III represents the type of fracture crack, and *r* and *θ* are distributed as the polar radius and rotation angle of the plastic zone at the crack tip.

*K*_*I*_ is used to represent the intensity factor of the stress field at the tip of I crack. Generally, it is considered that the calculation formula of I crack intensity factor is

(4) $$\begin{array}{c} K_{I}=\underset{r⟶0}{\lim}\sqrt{2\pi r}\sigma_{y}|{}_{\theta =0}. \end{array}$$

It can be seen that the stress distribution near the crack tip is a function of *r* and *θ*, which is independent of the load borne by the material or structure and the crack length. The general expression of stress intensity factor is

(5) $$\begin{array}{c} K_{I}=Y\sigma \sqrt{\pi \text{a}}, \end{array}$$

where *σ* is the nominal stress; a is the crack size; and *Y* is the shape factor. Literature review shows that the influencing factors of crack bend shape coefficient *Y* mainly include bend inner diameter, bend outer diameter, bend angle, and so on [9, 10].

## 3. Finite Element Modeling of Circular Through Crack Elbow

### 3.1. Structure and Geometric Parameters of Circumferential Through Crack Elbow

**Figure 1:**
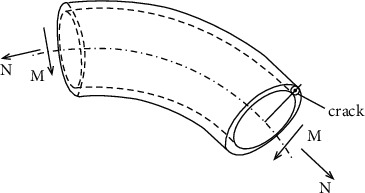
Cracked bend under tensile and bending load.

**Figure 2:**
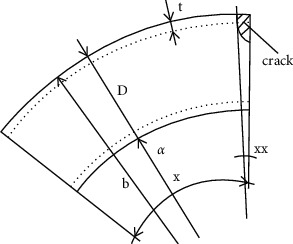
Structural dimension of elbow.

The common through elliptical crack elbow is selected as the analysis object. The geometric configuration and load of the crack in the elbow area are shown in Figure 1.  Figure 2 shows the structural dimension diagram of the elbow, in which a is the inner diameter of the elbow, *b* is the outer diameter of the elbow, *x* is the center angle of the crack, xx is the bending angle of the elbow, *t* is the wall thickness of the elbow, and *D* is the diameter of the elbow orifice.

### 3.2. Finite Element Modeling

**Figure 3:**
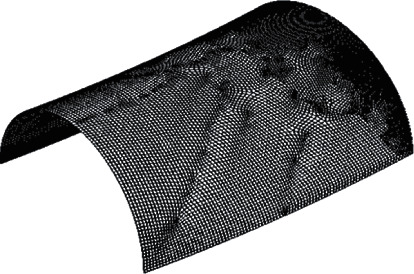
Network division diagram of elbow.

**Figure 4:**
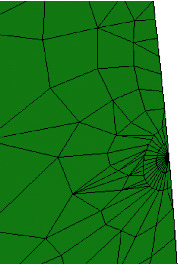
Finite element meshing diagram of crack tip with singular element.

To solve the crack stress intensity factor K by the finite element method, the three-dimensional model with crack must be established first. According to the characteristics of ANSYS finite element analysis [11], this paper establishes a symmetrical half bend model and completes the finite element meshing, as shown in Figure 3. The selected element is the shell element shell36 with 6 degrees of freedom, and the elastic modulus *E* = 2.1 × 10^−11^ and Poisson's ratio *μ* = 0.3. With the help of KSCON command, the crack tip meshing is established to generate the singular element of stress singularity at the crack tip [12], as shown in Figure 4.

## 4. BP Neural Network Design

### 4.1. BP Neural Network Learning Algorithm

**Figure 5:**
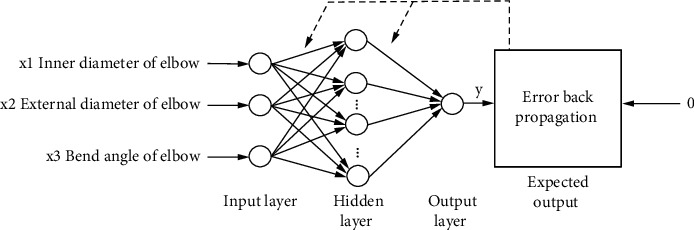
BP neural network model.

BP neural network is the neural network using the error backpropagation algorithm. Its algorithm is as follows: according to the negative gradient direction of the error between the actual output and the expected output of the neural network, the link weight between neurons of each layer is iteratively corrected layer by layer from back to front [13].  Figure 5 shows the three-layer BP neural network model designed in this paper. The learning steps of the neural network backpropagation algorithm are as follows [14]:

(1) Initialization: set all synaptic weights and thresholds to the minimum random number.
(2) Provide input training sample set {*X*_*i*_, *O*_*i*_}_*i*=1_^160^, where *X*_*i*_ = [*x*_*i*1_, *x*_*i*2_, *x*_*i*3_]^*T*^ and *O*_*i*_ = [*O*_*i*_] are used as input and output samples, respectively, and select learning step *η* = 0.6 to adjust the speed and seismic breaking degree of neural network searching the optimal weight. For *n* = 1, 2,…, 160 groups of samples were input circularly.
(3) For the training sample *n*, calculate the output of neurons in each hidden layer and output layer:
  (6) $$\begin{array}{c} y_{j}\left(n\right)=\phi_{i}\sum_{i=0}^{mL}w_{ij}y_{i}\left(n\right). \end{array}$$
(4) Calculate the error signal *e*_*j*_(*n*)=*o*_*i*_(*n*) − *y*_*i*_(*n*) and the cost function *ε*(*n*)=(1/2)∑_*j*=1_^*mL*^*e*_*j*_^2^(*n*).
(5) Adjust synaptic weights of output layer and hidden layer:
  (7) $$\begin{array}{c} w_{ji}\left(n+1\right)=w_{ji}\left(n\right)+\Delta w_{ji}\left(n\right),\\ \Delta w_{ji}\left(n\right)=\eta \frac{\partial \varepsilon \left(n\right)}{\partial w_{ji}\left(n\right)}. \end{array}$$
(6) Let *n* = *n* + 1 and return to step (3) until the stop criterion is met.

### 4.2. Topology of Prediction Intensity Factor Calculation Network

**Figure 6:**
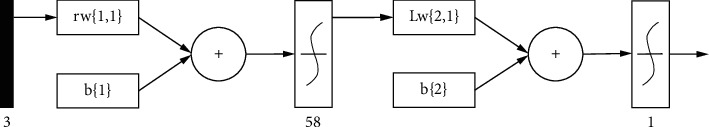
Network topology.

In this paper, a three-layer BP neural network structure is established, in which there are three nodes in the input layer. The input parameters are the inner diameter a of the elbow, the outer diameter *b* of the elbow, and the center angle *x* of the crack. The output is a node, that is, the intensity factor of the cracked elbow. The number of hidden layer nodes affects the accuracy of the network model. At present, there is no theoretical rule for its determination method. The number of hidden layer nodes is related to the amount of input and output information. The network topology is QG3—58—1, as shown in Figure 6. The “tansig” function is used as the activation function, the “purelin” function is used as the activation function in the output layer, and the “trainlm” algorithm is used for training.

## 5. Experimental Analysis

### 5.1. Obtaining Training Samples

According to the theory of fracture mechanics, the finite element analysis of the cracked elbow is carried out by ANSYS. The modeling size range is *a* = 79∼40 mm, *b* = 89∼50 mm; *x* = 2.8°; and xx = 10°∼15°. In this paper, a singular element is generated at the crack tip of the model, and the stress intensity factor is determined by the three-point displacement extrapolation method at the crack tip.

198 groups of neural network training samples were collected, 159 groups were used for neural network training, and 39 groups were used for neural network verification.

### 5.2. Result Analysis

#### 5.2.1. Network Training

**Figure 7:**
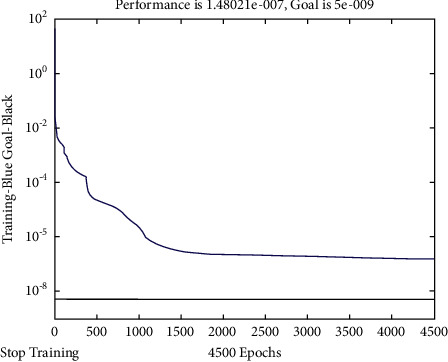
Error variation diagram of network training process.

In this paper, two methods, adding momentum term and adjusting learning rate, are used to improve the learning speed, increase the reliability of the algorithm, and avoid falling into local minimum during neural network training. Adaptively adjusting the learning rate shortens the learning time of neural network. In order to give better play to the performance of the training function, the training samples are quantified to the range of [−1, 1], and the error variation diagram of the network training process shown in Figure 7, as well as the connection weights of each layer and the threshold of each neuron of the network model shown in Table 1, is obtained. Through comprehensive analysis of Figure 7 and Table 1, it can be seen that the network training of this method reaches very high accuracy in step 4500, the mean square deviation of network output is about 2.7902 × 10^−7^, and the maximum fitting error is 0.0018, so the network output under the model in this paper is good for target tracking and has high prediction accuracy.

#### 5.2.2. Network Extension Test

**Figure 8:**
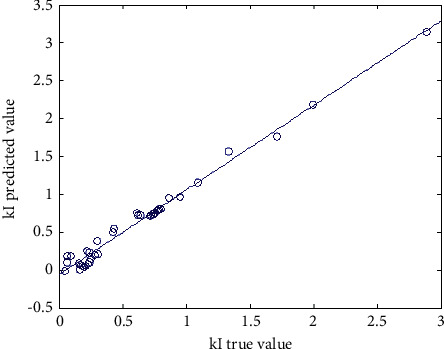
Linear regression analysis.

**Figure 9:**
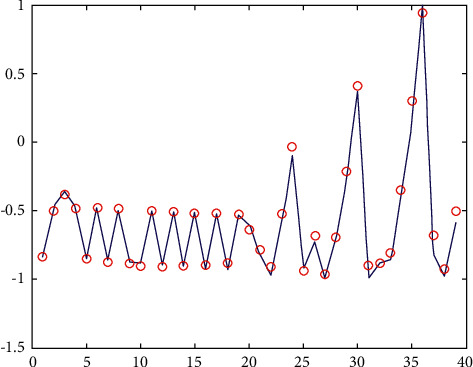
Network generalization tracking diagram.

**Figure 10:**
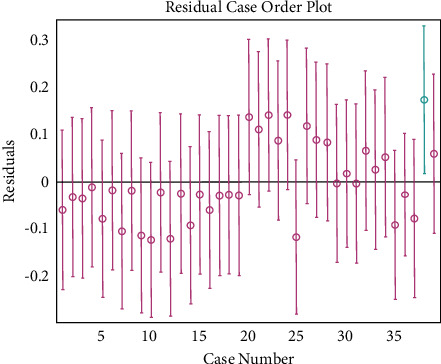
Residual diagram of neural network module ductility verification.

The prediction ability of the network to the samples not participating in the training is an effective method to evaluate the reliability of the model [15]. The root mean square error of the intensity factor *k* was 0.0102, the maximum error was 0.1544, and the average relative deviation of prediction was 0.19%.  Figure 8 shows the linear regression analysis diagram of the change rate of the network output change relative to the target value, the linear correlation determination coefficient is *R*^2^ = 0.9841, Figure 9 shows the network generalization tracking diagram, and Figure 10 shows the residual diagram of neural network model extension verification. According to the comprehensive analysis of Figures 8–10, the fitting degree between the change of network output and the change rate of target value is good, and the consistency between the predicted value of ANN model and the measured value is good. The error between the predicted value of network and the measured value is mostly distributed in the range of ±0.15, and the range of model error is appropriate. Therefore, the neural network prediction model has certain generalization ability and applicability.

## 6. Conclusion

In order to optimize the accuracy and generalization ability of the existing prediction model of stress intensity factor of circumferential through crack in elbow, a neural network model is introduced to optimize it, and the following conclusions are obtained:

1. The training samples are obtained based on the finite element analysis software, and the neural network prediction model of stress intensity factor of cracked elbow is established by using BP neural network. The relative error distribution of fitting and prediction is 0.000027% and 0.19%, which has high fitting and prediction accuracy in a large range.
2. Compared with the traditional method, the neural network method is used to establish the calculation model of stress intensity factor of through crack elbow structure, which can effectively avoid the difficulties of other prediction models and the errors caused by human factors.
3. BP network is used to replace the traditional calculation method, reduce the calculation difficulty, improve the calculation speed, and speed up the actual engineering structure design and structure analysis, so as to speed up the engineering process.

## Figures and Tables

**Table 1:** Network connection weights and thresholds.

Weight	Hidden layer node *j*					Weight	Hidden layer node *j*				
	1	2	3	4	5		6	7	8	9	10
Input node *i*	4.3785	−3.1792	2.4674	−1.1211	−3.8841	Input node *i*	1.0034	−0.9732	−3.8699	3.6769	−3.2183
	1.2491	−3.3175	2.8006	4.2375	1.6564		4.0752	2.5648	3.7926	−0.3202	−3.467
	−2.9388	−2.8731	3.929	3.1867	−3.3968		3.4284	4.6736	−0.0861	3.9681	−2.644
Output node *j*	0.4383	0.5581	−0.6342	0.5707	−0.9485	Output node *j*	−0.1852	0.8129	−0.3391	0.839	0.3792
Threshold *b*	−5.4192	5.2291	−5.0389	4.8488	4.6586	Threshold *b*	−4.4685	4.2783	4.0882	−3.898	3.7079
Weight	Hidden layer node *j*					Weight	Hidden layer node *j*				
	11	12	13	14	15		16	17	18	19	20
Input node *i*	−3.5057	−0.437	−0.7508	3.7164	2.6689	Input node *i*	3.09	−2.321	−2.1968	2.9501	5.1046
	−2.5833	−0.5516	−5.2312	2.7578	3.2876		−1.8204	4.7142	2.0777	2.2599	−1.1519
	−3.2256	−5.3733	1.1993	2.8197	3.3818		−4.0628	1.3257	−4.4972	3.9443	1.4087
Output node *j*	0.9053	0.7169	0.644	−0.0452	−0.2266	Output node *j*	0.9053	0.7169	0.644	−0.0452	−0.2266
Threshold *b*	3.5177	3.3276	3.1374	−2.9473	−2.7572	Threshold *b*	3.5177	3.3276	3.1374	−2.9473	−2.7572
Weight	Hidden layer node *j*					Weight	Hidden layer node *j*				
	21	22	23	24	25		26	27	28	29	30
Input node *i*	−3.4534	2.3038	−1.2402	4.7259	3.7538	Input node *i*	−2.9538	3.4927	3.542	−4.07	3.6032
	2.4541	−4.5069	0.1324	2.2871	−3.8169		−4.3738	1.1006	2.3476	−2.42	2.0621
	−3.3792	−1.9362	5.2738	−1.3426	−0.8414		1.2301	−3.9947	3.3632	−2.6357	−3.4832
Output node *j*	0.4937	−0.8105	−0.8095	0.8194	0.5549	Output node *j*	−0.7003	−0.7726	−0.5565	0.4276	−0.3155
Threshold *b*	1.6163	−1.4261	1.236	−1.0458	−0.8557	Threshold *b*	0.6655	−0.4754	−0.2852	0.0951	0.0951
Weight	Hidden layer node *j*					Weight	Hidden layer node *j*				
	31	32	33	34	35		36	37	38	39	40
Input node *i*	−1.9891	3.3707	−3.0278	−3.6975	−1.3709	Input node *i*	2.9496	4.0848	−0.5431	−1.8461	−3.8839
	1.8096	2.1345	−3.1601	3.9596	−5.2084		−1.8193	2.9899	4.0422	−3.2275	2.9865
	4.705	3.6674	3.196	0.1349	−0.6009		4.1663	−1.9346	3.5684	−3.9425	−2.316
Output node *j*	−0.5323	0.309	−0.4771	−0.1008	0.3014	Output node *j*	−0.6356	−0.0663	0.0656	0.2326	−0.1816
Threshold *b*	−0.2852	0.4754	−0.6655	−0.8557	−1.0458	Threshold *b*	1.236	1.4261	−1.6163	−1.8064	−1.9966
Weight	Hidden layer node *j*					Weight	Hidden layer node *j*				
	41	42	43	44	45		46	47	48	49	50
Input node *i*	−4.4054	−4.0902	4.1267	−0.8775	3.8594	Input node *i*	−3.8526	−0.5874	0.9973	−4.6247	0.6137
	2.9959	3.5286	−1.8715	2.5035	3.3894		−3.0495	2.2212	4.8828	1.2335	−3.7087
	−0.9926	0.4329	2.9725	−4.7255	−1.7276		2.286	4.9081	−2.1287	2.5414	−3.9035
Output node *j*	−0.178	0.3571	0.861	−0.9113	0.8466	Output node *j*	−0.1699	−0.9944	−0.3267	−0.5529	0.1583
Threshold *b*	−2.1867	−2.3769	2.567	−2.7572	2.9473	Threshold *b*	−3.1374	−3.3276	3.5177	−3.7079	3.898
Weight	Hidden layer node *j*					Weight	Hidden layer node *j*				Output node
	51	52	53	54	55		56	57	58		1
Input node *i*	2.2577	−2.4167	3.017	−5.3484	2.6632	Input node *i*	−2.6809	−1.3532	−4.0905		−0.1685
	3.6244	1.9388	0.1105	−0.6734	4.4392		−2.8943	−4.034	−1.7712		
	3.3369	4.4462	4.5004	−0.5559	1.6027		3.7154	−3.3562	−3.0819		
Output node *j*	0.5577	−0.4144	−0.5542	0.0968	0.728	Output node *j*	−0.0869	0.4252	−0.3411		
Threshold *b*	4.0882	−4.2783	4.4685	−4.6586	4.8488	Threshold *b*	−5.0389	−5.2291	−5.4192		

## Data Availability

The raw data supporting the conclusions of this article will be made available by the authors, without undue reservation.
